# Shared decision making in chronic kidney disease: a qualitative study of the impact of communication practices on treatment decisions for older patients

**DOI:** 10.1186/s12882-023-03406-9

**Published:** 2023-12-21

**Authors:** Maria R. Dahm, Suzanne Eggins Raine, Diana Slade, Laura J. Chien, Alice Kennard, Giles Walters, Tony Spinks, Girish Talaulikar

**Affiliations:** 1 Institute for Communication in Health Care (ICH), College of Arts and Social Sciences, Australian National University, Baldessin Precinct Building, 110 Ellery Crescent, Canberra, ACT 2601 Australia; 2 Canberra Hospital Renal Service, Canberra, Australia; 3 College of Health and Medicine, Australian National University, Canberra, Australia

**Keywords:** Physician-patient relations, Health Communication, dialysis, Shared-decision making, Qualitative research

## Abstract

**Background:**

Effective interpersonal communication is critical for shared decision-making (SDM). Previous SDM communication training in nephrology has lacked context-specific evidence from ethnographic analysis of SDM interactions with older patients considering treatment options of end stage kidney disease (ESKD). This study explores communication strategies in SDM discussions in nephrology, specifically focusing on older patients considering dialysis as kidney replacement therapy (KRT).

**Methods:**

We conducted a qualitative study analysing naturally-occurring audio-recorded clinical interactions (n = 12) between Australian kidney doctors, patients aged 60+, and carers. Linguistic ethnography and qualitative socially-oriented functional approaches were used for analysis.

**Results:**

Two types of communication strategies emerged: (1) Managing and advancing treatment decisions: involving active checking of knowledge, clear explanations of options, and local issue resolution. (2) Pulling back: Deferring or delaying decisions through mixed messaging. Specifically for non-English speaking patients, pulling back was further characterised by communication challenges deferring decision-making including ineffective issue management, and reliance on family as interpreters. Age was not an explicit topic of discussion among participants when it came to making decisions about KRT but was highly relevant to treatment decision-making. Doctors appeared reluctant to broach non-dialysis conservative management, even when it appears clinically appropriate. Conservative care, an alternative to KRT suitable for older patients with co-morbidities, was only explicitly discussed when prompted by patients or carers.

**Conclusions:**

The findings highlight the impact of different communication strategies on SDM discussions in nephrology. This study calls for linguistic-informed contextualised communication training and provides foundational evidence for nephrology-specific communication skills training in SDM for KRT among older patients. There is urgent need for doctors to become confident and competent in discussing non-dialysis conservative management. Further international research should explore naturally-occurring SDM interactions in nephrology with other vulnerable groups to enhance evidence and training integration.

**Supplementary Information:**

The online version contains supplementary material available at 10.1186/s12882-023-03406-9.

## Background

Worldwide, the number of patients with chronic kidney disease (CKD) facing decisions about kidney replacement therapy (KRT) is rapidly increasing, with the largest rise among older patients [1, 2]. Older patients in Australia are generally on dialysis treatment for a shorter period; patients starting haemodialysis at ages 65+, 75 + or 85 + years have a median survival time of 4.8, 3.6 and 2.6 years respectively [3]. After initiating dialysis, patients (including older patients) often express regret [4] and felt limited involvement in making the decision to start dialysis [5–7].

Evidence of poor outcomes and satisfaction for older patients raises questions about the shared decision making (SDM) processes around dialysis treatment [8, 9]. These concerns include whether information about suitable alternative non-dialysis conservative management is being presented [10, 11]. SDM has been variably defined and conceptualised [12–14], but generally requires at least two parties sharing information and taking steps to reach agreement on and implement a preferred treatment [14, 15]. Frequent and effective communication is crucial for SDM about KRT including dialysis [8, 16–18], especially for older patients who face greater uncertainty about prognosis and quality of life often linked to multiple co-morbidities [19, 20]. Currently, little formally structured communication training is available for nephrologists to acquire the skills necessary to successfully navigate these complex decision-making processes with their patients [4, 21].

Improved doctor preparedness has been reported for the few existing communication interventions aimed at enhancing doctors’ skills in having these difficult SDM discussions [22–25]. However, rather than drawing on an evidence-base of actual nephrology interactions, communication skills training for nephrologists (henceforth ‘doctor’) has been largely adapted from existing courses designed for other care specialities such as oncology and palliative care [21, 23] or combined with general concepts around breaking bad news [22, 24]. Using authentic interactions as a scaffold for contextualised communication skills training could further improve doctors’ engagement with patients, especially with respect to SDM [7]. Yet, to the best of our knowledge, no studies have analysed naturally-occurring interactions between doctors and patients with CKD to determine which communication strategies facilitate or hinder SDM. This study aimed to (1) identify doctors’ communicative practices during discussions with patients with CKD approaching decisions about KRT; and (2) determine how these practices impact on SDM.

## Methods

### Design, setting and participants

This study is part of a larger exploratory qualitative project [7] investigating the perspectives and communication strategies of doctors and nurses interacting with patients and carers during SDM, education and consent practices in the kidney outpatient clinic of a major metropolitan Australian teaching hospital. The study site provides care for the following patient populations: CKD stage 4/5 (n = 548), transplant (n = 242), dialysis (n = 340) and non-KRT (n = 20).

Local practice in relation to SDM involved doctors identifying patients with future KRT needs, briefly discussing different KRT options during consultations often at the same time or before referring them to a face-to-face nurse-led CKD education program [7]. In the weeks following education sessions, patients returned to their treating doctor and consultations further progressed SDM about KRT. Apart from one doctor, who completed palliative care related communication training, the doctors had not undergone dedicated training in SDM beyond what might have been included in their clinical and/or specialty education curriculum. The conversations occurred during a routine clinic review where the duration of each interaction was defined by the scheduling system and offered limited flexibility for expanding the duration of the session. Return visits for progressing SDM were limited by competing demands to provide access to new patients requiring kidney outpatient services.

Here, we present a close analysis of doctors’ communication practices in clinical interactions audio-recorded between September 2020 and February 2021. Australian travel and quarantine restrictions during the first year of COVID-19 meant, that following an initial lockdown period in the local jurisdiction, health care interactions proceeded largely as normal without mask requirements for face-to-face consultations during the data collection period. Telehealth consultations were offered but were not recorded for this study. CKD education sessions remained in-person but shifted from group settings to individual delivery.

We aimed to identify communication strategies during interactions related to patients with CKD deciding for or against starting dialysis. Thus, we sought to record as many different doctors addressing dialysis in routine consultations with patients with CKD as possible.

Eligible participants provided or received care at the study site. Following ethical approval (ACT Health Human Research Ethics Committees (2020.ETH.00040), MD, SER, DS and LC recruited a convenience sample of patients with advanced CKD (eGFR < 20 ml/min) aged 60 + who were currently involved in decision-making about dialysis and other forms of KRT. If present, carers (family members or caregivers) were also recruited. Clinical staff identified eligible patients, research team members (MD, SER, DS and LC) approached and consented patients and carers. Following a unit wide briefing session, MD, SER, DS and LC approached all doctors at the study site, and all but one participated. Reporting follows COREQ Guidelines (see supplementary material 1 for COREQ checklist).

### Data collection & analysis

Detailed data collection procedures are described elsewhere [7]. Here, we briefly sketch data collection and analysis pertaining to recorded clinical interactions.

Data collection days were scheduled to cover all doctors providing care at the site. Four qualitatively trained researchers (MD, SER, DS and LC) observed and audio-recorded 17 routine clinical interactions with patients with CKD not yet on dialysis; 12 interactions mentioned dialysis. The analysis presented here focused on these 12 interactions which were conducted by 5 out of 8 consented doctors. Researchers took detailed fieldnotes capturing situational context (e.g. seating arrangements) and nonverbal information (participant’s gaze or postures). All participants provided written informed consent. In line with ethical approval and to protect patient and doctor privacy, only basic demographic information was collected (Table 1).

**Table 1:** Participant demographics and duration of recorded routine clinical interactions (n = 12)

Participants (n = 27)	Female, n (%)	NESB, n (%)	Duration, range hh:mm:ss
Pre-dialysis patients with CKD^ (n = 11) Carers^#^ (n = 11) Doctors (n = 5)	4 (36) 5 (45) 1 (20)	3 (27) 3(27) 2 (40)	00.07.15–00.45.38

NESB: Non-English speaking background

^^^ one NESB patient was recorded in two separate visits

^#^two visits had no carer present, one visit had two carers present

Before analysis, we de-identified audio-recordings of clinical interactions during transcription; assigning each participant a code (patients with CKD: CKD-P1, doctors: Doc1).

SER followed ethnomethodology [26, 27] and systemic functional linguistics (SFL) [28] - a social-functional theory of language. SER, an SFL expert, checked the recordings and transcripts for completeness to closely familiarise herself with the data [29]. Applying social-functional discourse analytical techniques [26, 28, 30–32], and supported by situational information from fieldnotes, SER’s analysis focused on linguistic and discursive communication features related to SDM including: turn-taking (number of turns and how much the different participants talked); exchange structure (length and complexity of turns); participant communication roles (topic initiations, responses); and speech acts (e.g. clarifications, queries, statements, suggestions) [33, 34]. Analysis focused on the patients’ levels of knowledge and understanding of dialysis, as evidenced by their contributions, and the resolution of the interaction regarding dialysis (i.e. was a decision about dialysis treatment reached?).

We followed principles of qualitative data analysis [29] to safeguard rigour and trustworthiness in our analysis including prolonged engagement at the study sites, fieldnotes for contextual information and to cross-check initial interpretations, and triangulation of multiple perspectives (linguistic (MD, SER, DS, LC), clinical (AK, GW, GT) *and* patient (TS) views) in discussions of preliminary findings with the entire research team [35].

## Results

### Communication strategies affecting decision-making about treatment

Through observations and analysis of recorded interactions between doctors and patients with CKD not yet receiving dialysis treatment, we identified diverse communication strategies: those that advanced decisions and helped to manage difficulties in decision-making interactions, and strategies that deferred or delayed decision-making.

### Managing and advancing treatment decisions through effective communication

Three observed communication strategies were highly effective in advancing decision-making: (1) checking patient and carer’s disease and treatment specific background knowledge, (2) offering clear, specific and non-technical explanations of options and (3) locally managing the interaction to quickly resolve issues raised during the consultation.

### Active checking of patients’ and carers’ knowledge

Checking what the patient (and carers) already know about dialysis as a treatment option has obvious practical advantages. It allows doctors to tailor their explanations to the patient and carer’s level of knowledge while offering opportunities for them to be actively involved in knowledge-building – a necessary pre-requisite for decision-making. Excerpt 1 in Table 2 illustrates how the doctor recognised that the patient had a good initial grasp of peritoneal dialysis. The doctor explicitly asked the patient to ‘*summarise*’ (turn (t) 276) her understanding of ‘*dialysis via the tummy*’ (t274). The doctor forewent the more technical term ‘*peritoneal*’ which the patient subsequently used to signal both her understanding and to clarify (t275). Her admission of self-studying earned supportive laughter and positive evaluation from the doctor (ts278, t280 ‘*you’re using all the right terms*’). From turn 282, the doctor used the patient’s information to provide additional detail. The interaction was highly collaborative as doctor and patient jointly produced an explanation as evidenced by the patient beginning to pre-empt what the doctor was about to say (t287, t289, t291), before asking for one piece of information she wished to clarify (t293). This lay-friendly discussion of dialysis was efficient, focused and jointly constructed, indicating a high level of shared knowledge and shared purpose [14, 15, 36].

**Table 2:** Excerpt 1. Active checking of patients’ knowledge

Turn	Speaker	Talk
274.	Doc3:	What’s **your understanding** about **dialysis via the tummy**? Can you …
275.	CKD-P2	The **peritoneal**?
276.	Doc3:	Yeah, summarise it for me.
277.	CKD-P2:	Yeah. From what I’ve studied myself …
278.	Doc3:	**Good [laughs]**.
279.	CKD-P2:	… Dr Google, is that they—the access point is here in your stomach, in your abdomen. The fluid goes into the peritoneum and …
280.	Doc3:	Good, **you’re using all the right terms**. [to patient’s mother] She’s been studying very well [laughs].
281.	CKD-P2:	…in the peritoneum but if there is scar tissue in there it won’t cleanse that area properly, but that’s okay you can still do it, it will still do its job.
282.	Doc3:	Yeah. Yep. It’s based at home.
283.	CKD-P2:	Yeah.
284.	Doc3:	You have surgery…
285.	CKD-P2:	Yeah.
286.	Doc3:	…pre-emptively…
287.	CKD-P2:	**At an access point**. Yes.
288.	Doc3:	…so that we’re planning…
289.	CKD-P2:	**Set up. Yeah**.
290.	Doc3:	…ahead of your need for dialysis. The Tenckhoff catheter is inserted by the surgeon, generally needs a couple of weeks to mature before we…
291.	CKD-P2:	**Before they start to use it**.
292.	Doc3:	…start using it.
293.	CKD-P2:	**What about percentage? Do you get that done at 15 per cent or lower**?
294.	Doc3:	Generally, we start making arrangements around 15 per cent.
295.	CKD-P2:	Yeah, okay.
296.	Doc3:	But it’s also guided by your symptoms.

### Clear, specific and non-technical explanations of options

Because consultations are spoken interactions that unfold in real time, providing accurate, clear information in lay-friendly language without delay is a valuable communication skill [37–39]. In excerpt 2 (Table 3), following a clear and light-hearted discussion about peritoneal and haemodialysis, the doctor answered the patient’s question about transplants (t226) directly addressed the patient’s circumstances (t227 *‘for you’*) rather than give advice for a hypothetical patient. The doctor used clear and non-technical language (t227, t233, t237 *‘give you a kidney*’, ‘*downside to transplant*’, ‘*take a lot of drugs afterwards*’) avoiding opaque terminology such as ‘live donor’ and ‘deceased donor register’ that we observed in other consultations and education sessions [7]. The doctor also represented scientific evidence about transplant risks in concrete, everyday language that the patient grasped easily (t239, t241 *‘The risks to them are low, very low, but they’re not zero’, ‘actually do better […] do slightly worse’*) [36, 39]. This excerpt also illustrates the positive interpersonal relationships observed among the patient-doctor dyads, where patients (often in established, trusting relationships with their doctor) felt confident to change topics, interrupt and raise questions [39].

**Table 3:** Excerpt 2 Providing clear non-technical explanations

Turn	Speaker	Talk
226.	CKD-P7:	Oh. **What about a transplant**?
227.	Doc2:	So that’s the best option, and that would be the one that I would go for you, you know, because you don’t have to have dialysis. But you can’t have that before starting dialysis, unless somebody’s prepared to **give you a kidney**.
228.	CKD-P7:	Okay.
229.	DOC2:	So if someone, a friend, family, doesn’t really matter.
230.	CKD-P7:	Mm.
231.	DOC2:	You’re not allowed to advertise, but they don’t have to be that well-known to you. But if they’re willing to give you a kidney, then it can be organised, and we try to time it just before you’d otherwise need dialysis.
232.	CKD-P7:	Oh, okay.
233.	DOC2:	The **downside to transplant** is that you take a **lot of drugs afterwards**.
234.	CKD-P7:	Oh.
235.	DOC2:	To supress the immune system so that you don’t reject the kidney. And those drugs increase your risk of infection, significantly, particularly viral infections that you might not otherwise get.
236.	CKD-P7:	What about the other person?
237.	DOC2:	The person who’s given you a kidney?
238.	CKD-P7:	Mm.
239.	DOC2:	The **risks to them are low, very low, but they’re not zero**.
240.	CKD-P7:	Okay.
241.	DOC2:	If you look at people that have voluntarily donated a kidney compared to people who haven’t, the people who’ve donated **actually do better** than the people who haven’t donated a kidney. But that’s because we test them so carefully to make sure that they’re in really good health before they donate. If you actually compare the people that donate a kidney against people of a similar level of physical fitness and everything who haven’t donated, then the people that donate do **do slightly worse**.

### Well-managed interaction: the local resolution of matters raised

To effectively manage an interaction locally, doctors should ensure that issues raised by any party (patients, carers, doctors) in the interaction have been explicitly addressed and resolved before the consultation ends or acknowledge unresolved issues and schedule future interactions with intention of addressing them. Our analysis showed that in some, but not all, consultations, the doctors carefully managed the visit to achieve such local management. As a dialogue, medical consultations are, by nature, a turn-taking activity [40]. The minimal unit of interaction—*the exchange*—involves two turns of speaking, one by each participant, negotiating a single chunk of information or action. The quintessential exchanges are the question^answer pair or information^acknowledgement pair [41].

Excerpt 3 (Table 4) illustrates the prompt resolution of a question^answer exchange regarding the activation on a transplant waiting list being dependent on starting dialysis. The patient’s question (t668) was promptly answered (t669), and both parties acknowledged the response (t670, t671).

**Table 4:** Excerpt 3 A promptly resolved question^answer exchange

Turn	Speaker	Talk	Turn-taking activity
668.	CKD-P2	And then don’t that until you start dialysis, is that correct – for transplants?	Patient (Pt) asks question (q) 1
669.	Doc3	Generally.	Doctor (D) answers q1
670.	CKD-P2	Okay	Pt confirms understanding
671.	Doc3	Generally, that’s right.	D reiterates answer q1

Similarly, excerpt 4 (Table 5) shows the well-managed resolution of an exchange around the same topic involving question^answer and information^acknowledgement pairs. The doctors first answered (t284, t288, t294) each of patient’s questions (t283, t287, t293), before offering more information (t284, t286, t290, t292) which the patient acknowledged in (mostly) adjacent turns (t285, t289, t291, t295).

**Table 5:** Excerpt 4 A well-managed chain of question^answer and information^acknowledgment exchanges

Turn	Speaker	Talk	Turn-taking activity
283.	CKD-P7:	But so you can’t do a transplant after you’ve started dialysis, is it?	Patient (Pt) asks question (q) 1
284.	Doc2:	No, I’ve not been clear. So you can do it after you’ve started dialysis. What I had meant to get across is that you cannot have a transplant before dialysis unless somebody has come along to give you a kidney.	Doctor (D) answers q1 but then gives further clarifying information
285.	CKD-P7:	Oh, okay, okay.	Pt acknowledges information
286.	Doc2:	So if no one in your family and friends is giving you a kidney, then we have to start you on dialysis.	D gives further clarifying information to q1
287.	CKD-P7:	And then wait?	Pt asks q2
288.	Doc2:	And then activate you on the transplant waiting list.	D answers q2
289.	CKD-P7:	Yep, okay.	Pt confirms understanding
290.	Doc2:	And then you might, if you’re really lucky, get a kidney the next day.	D gives further clarifying information to q2
291.	CKD-P7:	Yeah.	Pt acknowledges information
292.	Doc2:	But more likely, you’ll wait, you know, three years.	D gives further clarifying information to q2
293.	CKD-P7:	Yep, and continue with dialysis until then?	Pt asks q3
294.	Doc2:	Yep.	D answers q3
295.	CKD-P7:	Yeah. Okay, all clear […].	Pt confirms understanding

However, well-managed interactions can also have more complex structures, as excerpts 5 and 6 (Tables 6 and 7) demonstrate. These excerpts, as all others, have been extracted from interactions with patients with CKD approaching KRT decisions. While not directly related to KRT or SDM, they serve to illustrate complex exchanges that have been successfully resolved locally.

**Table 6:** Excerpt 5. Turn-taking challenging local management of the interaction

Turn	Speaker	Talk	Turn-taking activity
283.	Doc7:	Are you getting any—have—have—have you checked the blood pressure between the fourth of November when you last saw me and today? Have you checked it in between with anyone?	Doctor asks question (q) 1
284.	CKD-P22:	No. [to carer] Ah, yes, didn’t I go to [unclear]?	Patient (Pt) answers q1 but then revises answer by asking carer q2
285.	CKD-P22’s carer:	Did they check your blood pressure when you were out there?	Instead of answering q2, asks q3
286.	CKD-P22:	I think so. You wouldn’t know out there. Well, I had to go there for something. What …	Responds to carer’s q3 but also begins to ask new q4
287.	CKD-P22’s carer:	Scripts wasn’t it?	Answers q4
288.	CKD-P22:	Scripts, yeah. He would have checked it surely. It’s the first thing they do, isn’t it?	Agrees with answer to q4, offers delayed answer to q1, asks q5
289.	Doc7:	I’d like to believe that that’s the first thing they do.	Responds to q5
290.	CKD-P22’s carer:	Not always, yeah.	Comments on Doctor’s response to q5
291.	Doc7:	Um, what we might need to do is restart you on one of the blood pressure tablets again […]	Dr returns to the issue that initiated the question sequence: the need to better control pt’s blood pressure

**Table 7:** Excerpt 6. Turn-taking in a locally managed interaction

Turn	Speaker	Talk	Turn-taking activity
23.	Doc7:	Um, are you able to describe the urinary symptoms a little bit more in detail? So what happens when you go to pass urine?	Doctor asks question 1 about symptoms
24.	CKD-P19:	Well, some—if—if I’ve got plenty of urine to pass, put it that way, um, it flows quite well. But as I—I’m trying to, um, articulate, when I started taking this Indapamide …	Pt starts to answer question, then changes topic to impact of medication
25.	Doc7:	So let’s—let’s keep—let’s—let’s put that aside because that’s actually building a cause and effect relationship, which is—which is not—not the intent. Just describe your symptoms to me. Just describe your symptoms. Let’s—let’s forget that—that it’s related to the Indapamide because—just—just describe what happens when you go to pass urine.	Doctor explains reasons to leave discussion of medication aside, returns focus to q1 multiple times
26.	CKD-P19:	Um, well, I—I think I indicated this is the first time I sort of—I didn’t have any real trouble passing urine … [provides further detail]	Patient answers q1

In a question^answer pair, as in excerpt 5 (Table 6), the doctor asked a question as the first turn of the pair ‘*have you checked the blood pressure between the fourth of November when you last saw me and today? Have you checked it in between with anyone?*’(t283). The patient provides the second turn of the pair answering the question initially with a simple ‘*no*’ (t284). However, sometimes a speaker cannot immediately resolve an exchange because they need further information first. Therefore, to resolve an issue, many exchanges involve three or more turns including follow-up comments, or checking/ clarifying turns by both participants. In excerpt 5 the patient then moved away from the original issue of exploring blood pressure fluctuations and initiated another series of exchanges with the carer (question^answer pairs; t284-290). After these tangential exchanges the doctor brought the negotiation back to focus on the original issue by suggesting to ‘*restart you on one of the blood pressure tablets*’ (t291).

Interactions can become structurally highly complex because they allow this insertion and addition of turn-taking activities within other turns in an open-ended chain (see excerpts 4 and 5 in Tables 5 and 6). In the dynamic context of spoken interaction, this means that participants may lose track of the starting point for a negotiation sequence [40, 41]. As in excerpt 5 (Table 6), one exchange can frequently be extended across multiple turns that negotiate the same piece of information or its components. If extended turns are not managed well, speakers may forget the purpose of the initiating turn—what was the question asked or the information offered? When this happens, participants can get ‘side-tracked’. Unanswered questions and unresolved matters provide linguistic evidence of ‘getting off track’.

Managing digressions, interruptions and irrelevancies is essential if doctors are to keep the interaction on track as illustrated in excerpt 6 (Table 7). Here the doctor interrupted the patient - going off track about the presumed role of one of his medications - to return to the original discussion of urinary symptoms.

Excerpt 6 shows that interactions and negotiations are tightly structured and effectively ‘locally managed’ when an exchange is resolved or completed in adjacent turns (i.e. as close to the initiating turn as possible) [40, 41].

In medical consultations, tight structure, with high levels of locally managed exchanges, are generally desirable because this structure ensures that questions are answered, advice is understood and next actions clearly stated in a logical, step-by-step sequence. In the consultation data, this type of structure was typical for several but not all doctors. As illustrated in excerpts 5 and 6, Doc7, for example, routinely used strategies to ensure that each parcel of information or set of issues was resolved before the talk moved on to another issue.

### Pulling back: Deferring or delaying decisions through communication strategies

In some interactions doctors stressed early-on that patients needed to decide and plan before ‘pulling back’ and deferring such decision-making. On occasions, doctors seemed to defer, not only encouraging participants to reach a decision (which may be entirely appropriate) but critically, also planning *how to progress* the discussion. We identified mixed messaging from doctors as a factor contributing to such deferred or delayed decision-making. Specifically, for NESB patients, mixed messaging often intersected with communication challenges deferring or delaying decisions: less effective local management of issues raised while using family of NESB patients as interpreters.

### Mixed messaging

Many doctors introduced the idea of planning for KRT including dialysis in relation to the patient’s current kidney function and symptoms by making clear and concise statements. In this discussion of acute kidney injury superimposed on CKD, the doctor’s clinical uncertainty about disease trajectory and prognosis disrupted clear messaging and decision-making:

*Yeah. Um, the kidney function is unchanged. So it’s still around 19 per cent. Like I said to you, we need to wait a few more-few more months, ah, you know, and decide on whether that is going to be your new normal or not.[ … ]. If you’re lucky and it improves then, of course, well and good. If it doesn’t then I will take you through, ah, what-what dialysis will look like and what you might choose to have. Doc7 to CKD-P19*.

*But predicting exactly when you need [a transplant] is hard, so we don’t want to leave it too late either […] But you don’t have to make any decisions now. Doc2 to CKD-P7*.

However, the level of intensity with which planning and decision-making was discussed (often in the face of uncertainty) before pulling back, made the difference between well-managed interactions and those with mixed messaging. Deferred decision interactions often contained mixed messaging; moments where doctors undercut any prior impetus to (potentially urgent) planning and decision-making discussions by assurances that patients did not need to decide ‘yet’. The desire to avoid overwhelming patients with information about treatment options sometimes led doctors to communicate mixed messages about the need for a decision or plan required actions. Excerpt 7 (Table 8) shows mixed messaging around the need for surgical access. After euphemistically declaring the patient’s kidney function as concerning (t36, t38 *‘sailing pretty close to the wind’*), the doctor recommended seeing a surgeon for dialysis access (t42, t44 ‘*So I think we should get you to see the surgeon’*). However, in the following turns (t46, t48), the doctor quickly retracted the suggestion declaring that they *‘can always delay the actual surgery’* (t48) if the patient’s kidney function improved.

**Table 8:** Excerpt 7 Mixed messaging in discussing surgical access for KRT

Turn	Speaker	Talk
36.	Doc2:	So I mean at 11 per cent function…
37.	CKD-P13:	Getting close to the…
38.	Doc2:	…**sailing pretty close to the wind**.
39.	CKD-P13:	…wind, yeah.
40.	Doc2	Yeah, um you have - have you seen the surgeon yet?
41.	CKD-P13:	No, no.
42.	Doc2:	So I think we **should get you to see the surgeon**.
43.	CKD-P13:	Right.
44.	Doc2:	Um, **to get the PD tube put in**.
45.	CKD-P13:	Right.
46.	Doc2:	Now, by the time you see the surgeon, a few weeks will have gone by…
47.	CKD-P13:	Right.
48.	Doc2:	…um, so we’ll have another blood test. If your kidney function did get better and it turned out that we didn’t need it, then **we can always delay the actual surgery**.

Pulling back from the need for vascular access surgery stood in contrast to other consultations in which doctors positively emphasised the level of preparedness afforded to patients with established surgical access:

*Yep, that looks good. Anyway, you don’t need it [the fistula] now […] so you know this is just more like an insurance uh, in case you need dialysis at some point. Doc1 to CKD-P17*.

*The kidney count has not changed much, it’s still 11 per cent. You don’t need to start dialysis and if you do need to that [fistula] is looking good. Doc7 to CKD-P18*.

### Communication challenges, and deferred decision-making in consultations with NESB patients

In effectively ‘locally managed’ interactions all issues raised by patients, carers or the doctors themselves were explicitly resolved by the end of the consultation. In less effectively ‘locally managed’ interactions, patients’ questions or issues remained unanswered or unresolved. This inconclusiveness often meant that SDM could not progress. Doctors may have more difficulty locally managing interactions involving NESB patients. In our data, NESB patients often attended consultations with younger family members (their children) as interpreters. Communicating through family members created issues related to time management, ambiguity about the patient’s knowledge of their disease and treatment options, and sometimes an apparent reluctance by the doctor to accept the patient’s decision. Interpreted interactions required more time than those where doctors interacted with patients directly, thus doctors occasionally tried to abridge discussions which further deferred decision-making.

Tables 9, 10 and 11 (excerpts 8 to 10) provide an example of the common difficulties in managing interpreted interactions with NESB patients described above. CKD-P18 was a native Korean speaker with limited English proficiency (excerpts 8 and 9, Tables 9 and 10). Her son facilitated communication with the doctor. Early in the 18-minute consultation the doctor asked the son whether the patient had agreed to a plan for treatment (t14). The son and the doctor briefly discussed the patient’s previously communicated reluctance to commit to dialysis. The son stressed his mother’s wish to see if dietary changes and alternative medicine would have an impact on her kidney function (t16-25 omitted). The son explained that the patient wanted to ‘*wait a little bit*’ (t26), while the doctor argued that the decision was needed fairly urgently (t32, t34 ‘*we need to start*’; ‘*so it’s good to actually plan now’, ‘get an AV fistula now’*). The son seemed unaware of the need for access surgery to prepare for dialysis. In contrast to excerpt 1 (Table 2), the doctors here did not initially check the patient’s or son’s knowledge or understanding of required access for dialysis. After explaining the need for a fistula (t30-50 omitted), the doctor again stressed the importance of making the decision *now* (t59 ‘*get the ball rolling now’*) by warning of the consequences of delay (t55t/57 ‘*which is not ideal’*).

**Table 9:** Excerpt 8. Urging a decision

Turn	Speaker	Talk
14.	Doc1:	The, um - so what was the plan essentially, in terms of when the kidney function goes down, because last time she was quite resistant to any - anything…
15.	CKD-P18’s son:	Yeah, I mean, she’s still quite reluctant, but um, she has um, had a visit to the, um, nutrition, um, management, um, at the [local Community Health Centre].
16–25 omitted		
26	CKD-P18’s son:	So she would like to see if, um, she could **wait a little bit to see**, you know, if any…
27.	Doc1:	See, I’m not saying you’re to start dialysis straight up.
28.	CKD-P18:	[Speaks Korean].
29.	CKD-P18’s son:	Aha.
30.	Doc1:	Okay, so what I’m saying is, when the kidney function reaches around eight or so…
31.	CKD-P18’s son:	Mm-hm.
32.	Doc1:	…**we need to** start dialysis, okay? So, she’s around 11, **so it’s good to actually plan now**, rather than in the last moment, scamper around, trying to find the mode of dialysis.
33.	CKD-P18’s son:	Mm-hm.
34.	Doc1:	Because, um, the ideal way to do it is to actually get an AV fistula **now**.
35–50 omitted		
51.	Doc1:	Now, if this is not done…
52.	CKD-P18’s son:	Mm-hm.
53.	Doc1:	…um, then if she needs dialysis and say, the next time you come for whatever reason it’s nine or eight or below…
54.	CKD-P18’s son:	Mm-hm.
55.	Doc1:	…then we’ll have to like, put in a line in her neck for dialysis, **which is not ideal.**
56.	CKD-P18’s son:	All right.
57.	Doc1:	**Which is not ideal**, I mean, we can do it, because when you put the line in the neck, um, it’s obviously a foreign body there, it’s prone to infections and other things, er, and then I’ll have to refer her for the fistula, which we are looking at - you’re on - dialysing through line for about four or five months at least.
58.	CKD-P18’s son:	Mm-hm.
59.	Doc1:	So if you get - **get the ball rolling now**, then maybe, you know, somewhere in February or March she can get the - get the surgery for the…

**Table 10:** Excerpt 9. Pulling back and sending mixed messages

Turn	Speaker	Talk
69.	Doc1:	But of course **I’m not going to**, you know, **force her** to have anything.
70.	CKD-P18’s son:	Hm.
71.	Doc1:	Um, it’s on **choice** and when it comes to it, it’s, you know, for dialysis they’ll just put in a and dialyse.
72.	CKD-P18’s son	Mm-hm.
73.	Doc1:	So, that’s the thing. She’ll definitely consider dialysis, is that?
74.	CKD-P18’s son:	Well, yes, um, haemodialysis, to be specific.
75.	Doc1:	Yes.
76–103 omitted		
104.	Doc1:	Yeah, I know. No, I - I mean I totally get what she’s saying, she - she’s a bit worried and scared and she wants to kind of postpone that, er, thing as much as possible. Everyone wants to postpone it, no one wants to get into dialysis straight up, um, and we will not start on dialysis, um, unless she needs it.
105.	CKD-P18’s son:	Hm, like eight, and say…
106.	Doc1:	And also, just because she has a AV fistula **doesn’t mean that she goes onto dialysis**.
107–121 omitted (mother and son conversing in Korean)		
122.	Doc1:	Because **I’m worried** that she’s—she’s—she’s lost weight not just because she’s, you know, suddenly started exercising or going on a diet, but **I’m worried** because of the kidney impairment that, you know is impacting her …
123–141 omitted		
142.	Doc1:	Okay, anyway, **it doesn’t matter**.
143.	CKD-P18’s son:	Sure.
144.	Doc1:	**There is no emergency** to start it **at the moment anyway**, um, it’s just that it would be good to, you know, prepare, that’s all, um…
145.	[Over speaking]	
146.	CKD-P18’s son:	That’s right, yeah. Well thanks for the, you know, heads up.
147–231 omitted		
232.	Doc1:	**But if you decide**, er, let, er, [first name kidney nurse] know…
233.	CKD-P18’s son:	Mm-hm.
234.	Doc1:	…**so we can organise the AV fistula**.
235.	CKD-P18’s son:	Sure.
236.	Doc1:	Okay, just because you get the [fistula] **doesn’t mean we’ll start dialysis** but at least…
237.	CKD-P18’s son:	That’s right.
238.	Doc1:	…get the ball rolling.
239.	CKD-P18’s son:	Mm-hm. I’ll make sure she understands that.
240.	Doc1:	Yeah.

**Table 11:** Excerpt 10 Mixed messages and less effective local management

Turn	Speaker	Talk
21.	Doc6:	So, she–you’re sitting about 10 per cent now, for your kidney function.
22–198 omitted		
199.	Doc6:	So, usually when people start reaching your kidney function, about 10 per cent, we start looking at dialysis, **really**.
200–346 omitted		
347.		It’s a possibility that, you know, obviously, she needs **time to think** about it. But in the meantime, that she might **deteriorate** in the meantime, and she might come to a situation where she needs an **emergency dialysis.**
348.	CKD-P8’ Daughter-in-law: (DIL)	Oh, okay.
349.	Doc6:	Yeah, so, it’s – obviously, you know, she’s thinking that **she doesn’t want to do it**.
350.	CKD-P8’ DIL	Yeah, that’s what the GP told me, that girl one. **She’s not happy now to do it**, when she will decide if to do it. But I’m - kidney level will be going down and down and that will a more painful for her.
351.	Doc6:	Hm.
352.	CKD-P8’ DIL	So, because she was keep telling me that **she has to do** dialysis when the **kidney level was 16**.
353.	Doc6:	Hm.
354.	CKD-P8’ DIL:	But my mother-in-law **didn’t make any mind to do it then**.
355.	Doc6:	Yeah, and she wouldn’t be the only one. I think a lot of patients find it’s hard to want to – it’s a very big change in life, isn’t it. You know, one day, you’re fine, the next day, you’re cooped up to – hooked up to a machine three days a week or, you know, every day and night. You have to go to more doctor appointments, you get blood tests, I mean, it’s going to be a big change for her. A lot of people do **take some time to come to terms** with that, especially when they are feeling quite okay. They say, why do I need dialysis? I feel okay.
356.	CKD-P8’s son:	Yeah, at the moment, she’s doing all right.
357.	Doc6:	Yeah. But we do know that this will **get worse** with time. So, you say, look, **let’s just pre-empt it and get all these things planned in advance**. Rather than wait for her to be **really unwell** one day, come to hospital **feeling really sick**, you know, her **unsafe** blood tests and things. But I know, it’s **obvious to me she has to think about it on her own terms**. **No-one should force her** into it.
358–375 omitted		
376.	Doc6:	So, look, for now, I won’t – **I don’t want to force you to decisions**, I just – my role is to give you an explanation of why we do things for her.
377–627 omitted		
628.	Doc6:	But we’ll do a blood test and any trouble, let us know in the meantime.
629.	CKD-P8’ DIL	Yeah. We’ll see if she can **make decision soon**.
630.	Doc6:	Yeah, I know…
631.	CKD-P8’s DIL	Yeah.
632.	Doc6:	… that it’s not an easy one to do for her and for you guys as well.
633.	CKD-P8’s DIL:	It is. It’s not an easy one to…

Excerpt 9 (Table 10) continues as the son updated his mother, the doctor started to pull back from urging a decision (t69) while still pushing for an answer (t73). Lengthy interpreted discussions revealed the patient’s preference to await the impact of acupuncture treatment, exercise and dietary regimes (t76-t103 omitted). The doctor did not offer evidence-based information on the likelihood of her kidney function continuing to decline or discuss alternative options for non-dialysis conservative management. Consequently, there is no “choice” as only one option is offered. Instead, the doctor offered an empathetic interpersonal reaction (t104), pulled further away from urging a treatment decision and explained that having a surgical access prepared did not mean starting dialysis straight away (t106). Later, the doctor interrupted the son and his mother (t107-121 omitted) to again highlight the urgency of the situation and his worry about the patient’s recent weight loss (t122). The mother via her son disagreed with the doctor assessments, discussing alternative treatment and current medication (t123-141 omitted). The doctor does not further enquire about the alternative treatment thereby leaving the matter locally unresolved. At this point (11 min into the interaction) the doctor seemed to decide there was no benefit in further discussion and moved to close the consultation without further pursuing a decision (t142 ‘*it doesn’t matter*’). The claim that ‘*there is no emergency* to start it *at the moment anyway*’ (t144) contradicts the previous urgency of the doctor’s explanations. The consultation continued with a physical examination, renewing prescriptions, and providing various blood test forms (t147-231 omitted). The doctor ended the consultation by repeating the pathway for fistula referral (t232, t234 ‘*But if you decide […] so we can organise the AV fistula*’) while also pulling back again from this being a definite decision for the patient to undergo dialysis (t236 ‘*just because you get the [fistula] doesn’t mean we’ll start dialysis’*).

Throughout this encounter (excerpts 8 and 9), the doctor sent mixed messages to the patient and son by first urging the patient towards a decision (t14, t32, t34, t59, t72, t122, t232, t234) before pulling back from such a decision (t69, t106, t142, t144, t236). The patient’s desire to see if alternative medicine, diet and exercise would affect her kidney function was not efficiently managed locally, as the doctor disengaged from discussing these topics. Finally, having to relay information to the patient or the doctor via the son took up large stretches of the 18-minute interaction, leaving little time for further in-depth discussion of unresolved issues. The consultation ended with the son promising the doctor to make his reluctant mother *‘understand*’ (t239), which begs the question of how well the patient’s voice, agency and her understanding of the situation was assessed and acknowledged in the interaction.

Excerpt 10 illustrates a similar pattern of mixed messaging and delayed planning and decision-making observed in another family-interpreted interaction (truncated in Table 11). Patient CKD-P8, of Indian descent with limited English proficiency, attended the consultation with her son, daughter and daughter-in-law. Initially, the doctor neutrally mentioned the patient’s current kidney function (t21) before later beginning to emphasise the need to *‘really’* (t199) discuss and plan for dialysis as a potential treatment. In a brief exchange with the doctor and CKDP-8’s daughter-in-law, they described the patient as reluctant to receive dialysis (t349, t350 ‘*she doesn’t want to do it*’, ‘*she’s not happy now to do it*’). The patient had not yet made a decision even though her daughter-in-law reported that the patient believed that she should decide once her kidney function declined to 16 per cent (t352, t354 ‘*she has to do dialysis when the kidney level was 16*’, ‘*didn’t make any mind to do it then’*). The assumed requirement for a decision is not attended to by the doctor and the matter of the patient’s assumption remains locally unresolved. While acknowledging the patient’s stance and time taken to reach a decision (t347, t355, ‘*time to think*’, ‘*take some time to come to terms with*’), the doctor stressed the need for a decision sooner rather than later (t357 ‘*let’s just pre-empt it and get all these things planned in advance’*) using increasingly serious and potentially scary language in the interaction (t347, t357 ‘*deteriorate*’, ‘*emergency dialysis*’; ‘*get worse with time’*, *‘really unwell*’, ‘*feeling really sick*’, ‘*unsafe*’). Simultaneously, the doctor started pulling back and emphasised that the decision was ultimately the patient’s (t357, t376 ‘*no-one should force her’*, ‘*don’t want to force you to decisions*’). Similar to CKD-P18 (Tables 9 and 10), the interaction ended without a clear decision or a specific timeframe (t629 ‘*soon*’) for a decision. Once again, there was no discussion here about the role and possibility of non-dialysis conservative management, so no real choices were offered.

### Age as a factor in interactions

While this study focused on older patients with CKD approaching KRT decisions, age was not an explicitly featured factor in relation to deciding about KRT in most observed interactions.

There was only one extended exchange focused on age in relation to a kidney transplant as a KRT option (Excerpt 11 Table 12). Yet, here the relevance of age was associated with the age of the kidney donor (live or deceased) rather than the patients’ age. In a trusting and collaborative interaction, the doctor and patient discussed the pros and cons of a younger vs. older donor and live vs. deceased kidney transplant (t368/70, t376 ‘*Young […] is better for you*’, ‘*A living kidney, if you’re considering, do I want it from him, he, as you say, is old, compared to waiting for a deceased donor, a living kidney is almost always better*’). The interaction then turned to the intricacies and impact of live kidney donation from younger and older family members. The patient stated she prefered her husband as a donor (t373 ‘*he’s old*’) which the doctor acknowledged whilst also highlighting that because of his age her son’s ‘*kidney is likely to be better for you than your husband’s because he’s going to be younger*.’ (t386). The patient restated her reasoning to ‘*leave the young ones alone*’ (t389) which the doctor acknowledged before presenting a ‘*backup*’ (t392) option.

**Table 12:** Excerpt 11 Discussion of age in relation to kidney donors

Turn	Speaker	Talk
367.	CKD-P7	Is it better to get a kidney from a **young person or an older person**?
368.	Doc2:	**Young**.
369.	CKD-P7 :	Is it?
370.	Doc2:	Better for you.
371.	CKD-P7 :	Oh. I was thinking maybe [John(husband)] can give me one of his. My husband. [Laughs]
372.	Doc2:	Yeah, sure, if he wants to.
373.	CKD-P7 :	But **he’s old**.
374.	Doc2:	How old is he?
375.	CKD-P7 :	He’s three years older than me, four years older than me.
376.	Doc2:	That’s okay. A living kidney, if you’re considering, do I want it from him, he, as you say, **is old**, compared to waiting for a deceased donor, a living kidney is almost always better than one from someone who’s died.
377.	CKD-P7 :	Okay.
378.	Doc2:	Because it’s taken out from them in a controlled fashion, and then it’s put straight into you.
379.	CKD-P7 :	Yep.
380 omitted		
381.	CKD-P7 :	What about the risk of rejection, [John(husband)] or [Charlie(son)], it would be better for me to adapt? Because [Charlie(son)] is like…
382.	DSS02:	Well, [Charlie(son)] is likely to be a better match.
383.	Patient N:	Better match.
384.	Doc2:	Yeah. But we don’t yet know whether you have any antibodies. You may from pregnancy.
385.	CKD-P7 :	Mm.
386.	Doc2:	So we don’t know how hard that side of things would be. [Charlie(son)]’s kidney is likely to be **better for you than your husband’s because he’s going to be younger**.
387.	CKD-P7 :	Mm.
388.	Doc2:	But I think either would be pretty good.
389.	CKD-P7 :	Okay, because I’m just thinking, **leave the young ones alone**. We can grow old together with one kidney each. [Laughs]
390.	Doc2:	It’s a reasonable way to think. And also, if that kidney didn’t work out…
391.	CKD-P7 :	Then I’ve still got [Charlie(son)]. [Laughs]
392.	Doc2:	You’ve still got [Charlie(son)] as a backup.
393.	CKD-P7 :	[Laughs]
394.	Doc2:	It’s tough, isn’t it?

Studies on older patients with CKD have shown that dialysis does not necessarily afford them the desired quality of life, leading these patients to adopt conservative care dedicated to symptom management [10, 11, 42]. Local education materials included non-dialysis conservative care as a management option and two supportive care clinicians (a doctor with nephrology palliative care training and a nurse) were available on staff. Yet, in our data, conservative care was only mentioned when patients (or carers) specifically asked about it:

If I don’t do the dialysis, what’s happening to me? CKD-P16 to Doc1

*If she doesn’t want to do dialysis at all [.] because GP told me that she would have only three to six months for living life. CKD-P8’s DIL to Doc6*.

CKD-P8’s doctor briefly explained the options of conservative care (mislabelled as supportive care indicating a lack of familiarity with non-dialysis options) without mentioning death. The doctor again sent mixed messages stating that even if the patient chose conservative care, dialysis could still be started if she changed her mind:

*As her kidney function starts declining slowly, we have the option of offering what we call supportive care, which means the patient has said that I don’t want to have dialysis for sure, I don’t want–I don’t want haemo, I don’t want [PD], I don’t want anything. Then we focus on trying to manage her symptoms, you know, make sure her–give as much medication as possible so, you know […] If she’s feeling sick all the time, we can give her something to control the nausea. But it’s not a one-way trip, you know. If she half-way says, look, I’m–I’ve gotten so sick, I think I want dialysis now, we’re still happy to provide it. Doc6 to CKD-P8 and carers*.

For CKD-P16, on the other hand, neither conservative care, related symptom management, palliative care nor death was directly addressed:

*Well, if you don’t do dialysis when um, below a particular number, below six to eight per cent… below six per cent, ah then you can be more sicker, um you won’t feel like eating anything…you’ll feel short of breath. […] if you go on to dialysis, um at that particular time…if you decide okay I don’t want to, um, then that’s the–that’s the only option that you’ll have. Doc1 to CKD-P16*.

Similarly, on one occasion when a doctor described what would happen if a patient decided against KRT (without the patient prompting), the concept of conservative care and symptom management was not directly addressed, even though death was:

*Now, if you did get to the point where you were getting quite sick because of the kidney disease, and you chose not to have dialysis, or we didn’t get a kidney transplant for you, then the symptoms would continue. You would get more and more tired, and you’d fade away and die. So most people choose to have dialysis. Doc2 to CKD-P7*.

## Discussion

In this study, we analysed nephrology consultations and identified communication strategies that can drive or delay SDM about KRT among older patients with CKD. To advance SDM, doctors checked patients pre-existing knowledge, provided lay-friendly tailored information and resolved questions and issue within the same consultation. SDM was disrupted when doctors sent mixed messages about the need for a decision (including pulling back on previously expressed urgency to make a decision), declined or avoided discussions about conservative non-dialysis management and for interactions with NESB patients left questions or concerns unresolved and faced the complexity of using family as interpreters. When doctors encourage patients to decide on a course of action but then pull back, they undercut the initial sense of urgency they created and may cut short further discussion. This can lead to deferred decision-making. It is understandable that patients might not feel able to decide on the spot and that doctors may face clinical or prognostic uncertainty. For complex decisions, where possible, SDM should be an iterative process with time and multiple opportunities and invitations for patients, carers and doctors to clarify information and reconsider their options [8, 9]. SDM is iterative and if decisions remain unresolved in an initial encounter, a plan should be made to facilitate future resolution. Such effective SDM communication can be one of the greatest challenges of managing chronic progressive diseases in resource-limited fragmented healthcare settings focussing on episodic care that may not be in keeping with the changing burden of disease [43–45]. Our findings on mixed messaging suggest that it can be difficult for doctors to find the right balance between planting seeds to start this iterative process and using language that conveys urgency and/or uncertainty. Mixed messaging can cause confusion among patients but also indifference and complacency rather than engagement and agency in SDM.

Our evidence showed a variety of challenges doctors face moving some patients towards decision-making, given the clinical, cultural, linguistic and health literacy complexities of the context. Doctors engaging in initial as well as ongoing SDM communication with patients considering (or receiving) KRT, should receive specifically targeted training to provide them with practical communication strategies and protocols to inform and negotiate timely decision-making. Training should also cover instruction on asking patients how they wish to receive information about death and facilitating open discussions about conservative care, death and dying. Doctors should seek to become familiar with supportive care and symptom management strategies and how these can be implemented in conservative management to increase their confidence and competence in discussing and offering non-dialysis conservative care. The sparse current communication training programs for nephrology include sample communication statements for doctors, but it is unclear how these training programs and sample statements were developed and if they were based on interactions from oncology, nephrology or generic breaking bad news communication training programs [21–24]. Adapting the use of best-case/ worst-case scenarios from surgery to nephrology has shown promise [46, 47], but studies remain more focused on the content of SDM communication (what is said) rather than its delivery (how it is said). Simply reporting that doctors mention the consequences of not choosing dialysis does not provide insight into the quality of their communication. By analysing naturally-occurring nephrology interactions, our study provides the first foundational evidence to contribute to contextualising nephrology-specific communication skills training surrounding SDM for KRT among patients with CKD. Future research should include analytical approaches from linguistics and health communication in the analysis of recorded interactions as they can provide the missing insights into the quality of SDM communication.

Our study also highlighted the challenges doctors face in interacting with patients from highly diverse cultural, linguistic and educational backgrounds and negotiating complex clinical situations within the constraints of the public health system. Using naturally-occurring data allowed us to trace communication encounters involving NESB patients and their carers, emphasising the communicative complexity involved in family-interpreted SDM interactions. Despite the additional barriers patients from culturally and linguistically diverse backgrounds face [48–50], they are often deliberately excluded from healthcare communication studies [51, 52] including patients with CKD [50, 53]. Without help from English-speaking carers to understand medical information, SDM for NESB patients can be hindered [48, 49]. Available under-utilised interpreter services should be mobilised to improve understanding and SDM, and doctors’ reasons for avoiding formal interpreters need to be explored further. Our study provides a first look into the structure of interpreted SDM interactions and provides crucial evidence into how multiple communication strategies that impede SDM might overlap in these consultations.

While our study focused on older patients with CKD, age was very rarely mentioned and even less likely to be addressed as a deciding factor for patients and doctors when discussing KRT which would ideally also require explicit discussions and consideration of comorbid status and functional status [11, 42]. Effective and informed SDM warrants explicit discussion of all clinically relevant factors and KRT options. Conservative care, an alternative to KRT suitable for older patients with co-morbidities [10, 11], was only explicitly discussed when prompted by patients or carers. Several doctors demonstrated reluctance to explore conservative management options, and their limited discussion revealed lack of familiarity with supportive care and symptom management strategies. These knowledge gaps likely reflect the low numbers of conservatively-managed patients cared for by this nephrology service, despite the presence of an established supportive care service. Our study’s findings may be constrained by our recruitment of older patients aged over 60 years as one group, potentially limiting our ability to observe more detailed age-related effects on SDM within this small qualitative sample. Future research should consider further and finer age stratification among older patients with CKD in recording larger samples of naturally occurring interactions approaching decisions about KRT. The study did not explore the impact of the broader service design and the impact of resource limitations on clinician approaches.

## Conclusion

This qualitative study combined ethnographic and linguistic research to build the first evidence-based catalogue of communication strategies used by kidney doctors in SDM discussions with older patients. While our study is limited to the communication practices of a small group of Australian doctors, it is the first ground-breaking step towards establishing an evidence-base of contextualised communication strategies to inform essential SDM and communication curricula in nephrology [4, 21, 23]. Opportunities to improve SDM practices include further education and familiarisation with symptom and supportive management and development of experience in offering conservative management. Further international exploration of communication strategies in nephrology interactions with more fine-grained stratification for age and functional status and larger qualitative samples is needed to ensure effective SDM to involve and support older and other vulnerable patient groups making life-altering decisions about KRT. The impact of health service design and resource limitations on communication approaches also needs further consideration.

### Electronic supplementary material

Below is the link to the electronic supplementary material.

Supplementary Material 1

## Data Availability

The data underlying this article cannot be shared publicly due to confidentiality and ethical requirements.

## Abbreviations

CKD: Chronic kidney disease
eGFR: Estimated glomerular filtration rate
ESKD: End stage kidney disease
KRT: Kidney replacement therapy
NESB: Non-English speaking background
SDM: Shared decision-making
SFL: Systemic functional linguistics
