# Supplementary material for: Shared decision making in chronic kidney disease: a qualitative study of the impact of communication practices on treatment decisions for older patients
Source: BMC Nephrol. 2023 Dec 21;24:383. doi: 10.1186/s12882-023-03406-9 (PMC10734071; doi:10.1186/s12882-023-03406-9)
Supplement: Supplementary file 1 — Supplementary Material 1 [file 12882_2023_3406_MOESM1_ESM.docx]

Table 1. COREQ (COnsolidated criteria for REporting Qualitative research) Checklist

| **No Item** | **Description** |
| --- | --- |
| Domain 1: Research team and reflexivity | |
| Personal Characteristics | |
| 1. Interviewer/facilitator | See page 5, line 18 & 22; field recordings and observations |
| 2. Credentials | MD – MA, PhD  SER – BA(Hons), PhD, MA(ProfComm), MLitt DEA, GDipCarCounsel  DS - BA, GDipEd, MA, PhD  LC – BA/BAs(Hons), GDipProfWrit  AK – BSc, MBBS(Hons), FRACPt  GW – MB ChB, MRCP(UK), MD FRACP  TS – BA, GradDip Ed, MA  GT – MBBS, FRACP, Grad Cert HE, M Clin Epid, Grad Cert HM |
| 3. Occupation | MD – Senior Research Fellow  SER – Research Fellow  DS - Professor of Applied Linguistics  LC – Senior Research Officer  AK – Nephrologist  GW – Nephrologist  TS – Consumer representative  GT – Director of Renal Services and Nephrologist |
| 4. Gender | Ability, skill and research quality are not related to a researcher’s gender. |
| 5. Experience and training | The researchers conducting the field recordings and observations were experienced qualitative researchers. |
| Relationship with participants | |
| 6. Relationship established | See page 5, line 22 & 23; Briefing sessions |
| 7. Participant knowledge of the interviewer | See page 5, line 22 & 23; Briefing sessions |
| 8. Interviewer characteristics | See page 5, line 22 & 23; Briefing sessions |
| Domain 2: study design | |
| Theoretical framework | |
| 9. Methodological orientation and Theory | See page 7, line 1-11 |
| Participant selection | |
| 10. Sampling How were participants selected? | See page 5, line 17-22 |
| 11. Method of approach How were participants approached? | See page 6, line 4 |
| 12. Sample size | See page 6, Table 1 |
| 13. Non-participation | See page 5, line 23 |
| Setting | |
| 14. Setting of data collection | See page 6, line 4; clinic |
| 15. Presence of non-participants | No |
| 16. Description of sample | See page 4, lines 19-20; page 6, lines 18-21 and page 6 Table 1 |
| Data collection | |
| 17. Interview guide | N/A |
| 18. Repeat interviews | N/A |
| 19. Audio/visual recording | See page 6, lines 4-7 |
| 20. Field notes | See page 6, lines 8-9 |
| 21. Duration | N/A |
| 22. Data saturation | N/A |
| 23. Transcripts returned | N/A |
| Domain 3: analysis and findings | |
| Data analysis | |
| 24. Number of data coders | See page 7, lines 1-7, 13-15 |
| 25. Description of the coding tree | N/A |
| 26. Derivation of themes | See page 7, lines 3-8 |
| 27. Software | N/A |
| 28. Participant checking | See page 7, line 13-15; triangulation with clinician researchers |
| Reporting | |
| 29. Quotations presented | See pages 7-22 and Table 2-12 |
| 30. Data and findings consistent | See pages 7-22 and Table 2-12 |
| 31. Clarity of major themes | See pages 7-22 and Table 2-12 |
| 32. Clarity of minor themes | No |

Based on: Tong A, Sainsbury P, Craig J. Consolidated criteria for reporting qualitative research (COREQ): a 32-item checklist for interviews and focus groups. International Journal for Quality in Health Care. 2007. Volume 19, Number 6: pp. 349 –357
